# Predictive Biomarkers of Pathological Response to Neoadjuvant Chemoradiotherapy for Locally Advanced Soft Tissue Sarcomas

**DOI:** 10.3390/cancers15112960

**Published:** 2023-05-29

**Authors:** Anna Szumera-Ciećkiewicz, Klaudia Bobak, Mateusz J. Spałek, Kamil Sokół, Michał Wągrodzki, Daria Owczarek, Monika Kawecka, Beata Puton, Hanna Koseła-Paterczyk, Piotr Rutkowski, Anna M. Czarnecka

**Affiliations:** 1 Department of Pathology, Maria Sklodowska-Curie National Research Institute of Oncology, 02781 Warsaw, Poland; szumann@gmail.com (A.S.-C.);; 2 Diagnostic Hematology Department, Institute of Hematology and Transfusion Medicine, 00791 Warsaw, Poland; 3 Department of Soft Tissue/Bone Sarcoma and Melanoma, Maria Sklodowska-Curie National Research Institute of Oncology, 02781 Warsaw, Poland; klaudia.bobak@pib-nio.pl (K.B.); mateusz@spalek.co (M.J.S.); piotr.rutkowski@pib-nio.pl (P.R.); 4 1st Department of Radiotherapy, Maria Sklodowska-Curie National Research Institute of Oncology, 02781 Warsaw, Poland; 5 Department of Experimental Pharmacology, Mossakowski Medical Research Institute, Polish Academy of Sciences, 02106 Warsaw, Poland

**Keywords:** predictive biomarkers, pathological response, neoadjuvant chemoradiotherapy, soft tissue sarcomas, immunohistochemistry, HIF1α, tumor-infiltrating macrophages, tumor microenvironment, γH2AFX

## Abstract

**Simple Summary:**

Soft tissue sarcomas (STS) are a large group of heterogeneous mesenchymal neoplasms. There is no standard treatment for STS and locally advanced, marginally resectable primary STS remain a treatment challenge for clinicians. Identification of a molecular biomarker of the pathological response (PR) would aid in the diagnosis and treatment of this group of patients. However, the molecular biology and genetic profile of STS are still poorly understood. The study aimed to identify a biomarker for PR prediction after neoadjuvant treatment in STS. We have chosen six markers (HIF-1α, CD163, CD68, CD34, CD105, γH2AFX) for immunohistochemical staining. We found a negative correlation between the expression of HIF-1α and PR, which means poor response to therapy. Furthermore, our results showed that a high expression of γH2AFX before treatment was positively correlated with PR, providing a putative biomarker of the response to treatment.

**Abstract:**

Background: Marginally resectable and unresectable soft tissue sarcomas (STS) remain a therapy challenge due to the lack of highly active treatment. The aim of the study was to identify a biomarker to predict the pathological response (PR) to preplanned treatment of these STSs. Methods: In the phase II clinical trial (NCT03651375), locally advanced STS patients received preoperative treatment with a combination of doxorubicin-ifosfamide chemotherapy and 5 × 5 Gy radiotherapy. PR to the treatment was classified using the European Organization for Research and Treatment of Cancer–Soft Tissue and Bone Sarcoma Group recommendations. We have chosen HIF-1α, CD163, CD68, CD34, CD105, and γH2AFX proteins, rendering different biological phenomena, for biomarker study. Results: Nineteen patients were enrolled and in four cases a good PR was reported. The high expression of HIF-1α before surgery showed a negative correlation with PR, which means a poor response to therapy. Furthermore, the samples after surgery had decreased expression of HIF-1α, which confirmed the correlation with PR. However, high expression of γH2AFX positively correlated with PR, which provides better PR. The high number of positive-staining TAMs and the high IMVD did not correlate with PR. Conclusions: HIF1α and γH2AFX could be potential biomarkers for PR prediction after neoadjuvant treatment in STS.

## 1. Introduction

Locally advanced, marginally resectable primary soft tissue sarcomas (STS) require intensive preoperative treatment before limb-sparing or conservative surgery [1]. Therefore, unresectable and marginally resectable sarcomas remain a treatment challenge for clinicians due to the lack of standard highly effective targeted treatment. At this point, most patients are treated with anthracycline-based neoadjuvant chemotherapy and/or radiation therapy [1,2]. While chemoradiotherapy has substantial toxicity, are still no predictive biomarkers predict the response to neoadjuvant STS treatment that are not well defined. Identifying a molecular biomarker of the pathological response (PR) will aid in personalized treatment selection. Currently, predictive markers of STS radiation therapy response include DNA damage repair genes, hypoxia signalling pathway genes, and tumor angiogenesis genes [3]. Until now, Affymetrix Hu-RSTA-2a520709 microarray—that provides data on 25,000 genes—was used to define a gene expression-based radiosensitivity index to identify radioresistant subsets of STS [4]. At the same time, RNAseq data for soft tissue sarcoma from The Cancer Genome Atlas (TCGA) were used to define a radiosensitivity biomarker. As a result of the analysis, 65 genes were selected as the radiosensitivity signature [5]. Moreover, it is known that complete pathological response (pathCR) is a predictor of a favorable long-term outcome in STS patients who are treated with pre-operative radiation (RT) alone [6]. Nevertheless, biomarkers of multidisciplinary treatment are not defined. 

Currently, the molecular biology and genetic profile of STS are generally not fully understood [7,8,9,10]. In STS, the genes that are most frequently mutated are *TP53* (in 47% of cases), *CDKN2A* (in 22%), *RB1* (in 22%), *NF1* (in 11%), and *ATRX* (in 11%). The most recent study, in a group of 1162 patients with sarcomas, identified mutations in *BRCA2*, *ATM*, *ATR*, and *ERCC2* genes [11]. Based on a study of 2138 sarcomas of 45 pathological subtypes, the most common mutations are found in cell cycle control genes, *TP53*, receptor tyrosine kinases/PI3K/RAS, and epigenetic regulators. *TERT* amplification is typical in intimal sarcoma, while SWI/SNF alterations are typical for uterine adenosarcoma [12]. At the same time, there are no biomarkers to predict the potential effectiveness of chemotherapy and radiotherapy for sarcomas (soft tissues and bones) [10,13]. Molecular predictive markers for sarcoma patients are also necessary to stratify patients and identify those who could benefit from more intensive therapeutic strategies [14,15], including neoadjuvant therapies such as those proposed in a phase II clinical trial (NCT03651375) reported by the authors of the present study [16]. A published biomarker study, RTOG 9514, evaluated the expression of proteins, such as CAIX, GLUT1, PARP1, and p53, before and after multidisciplinary treatment. In this study, the expression of CAIX, GLUT1, and PARP1 decreased significantly after neoadjuvant therapy. In contrast, the accumulation of p53 in the cell nucleus relative to the cytoplasm increased numerically, but no significant association was found with patient survival. Changes in expression pattern after neoadjuvant chemoradiotherapy described in this study support the concept of tumor reoxygenation, changes in HIF-1α-dependent signaling, and indicate activation of the DNA damage response pathway [17]. On the other hand, studies that evaluate radiation therapy (RT) revealed a 26-gene signature that allows the identification of patients with a good response to the treatment [18].

Due to the insufficient availability of data on the molecular basis of the development and response to STS treatment, we proposed six markers for immunohistochemical staining. Hypoxia-inducible factor 1-α (HIF1α) expression was selected as a biomarker of intratumoral hypoxia. The expression of the phosphorylated form of γ-H2A histone family X (γH2AFX) was detected to assess double-strand DNA breaks, which could be caused by radiation and chemotherapy. As biomarkers for the tumor microenvironment (TME), CD163 and CD68 (tumor-infiltrating macrophages—TAM), as well as CD34 and CD105 (intratumoral microvascular density—IMVD), were chosen.

The study aimed to identify a biomarker to predict the pathological response to STS treatment. We hypothesized that PR could be predicted using biomarkers of TME, hypoxia, and DNA damage.

## 2. Materials and Methods

### 2.1. Characteristics of the Patient

Adult patients (>18 years) with locally advanced, marginally resectable STS of the extremities or trunk wall were recruited to our prospective open-label single-arm Phase II clinical trial (NCT03651375). The inclusion criteria and treatment have been previously described [16,19,20]. All patients provided written informed consent for the study.

### 2.2. Pathological Response

The evaluation of pathological response (PR) was conducted as per recommendations of the European Organisation for Research and Treatment of Soft Tissue and Bone Sarcoma Group (EORTC–STBSG) [21] with: A type response—no stainable tumor cells, while B—single stainable tumor cells or small groups (overall below 1% of the whole specimen), C—1%–<10% stainable tumor cells, D—10%–<50% stainable tumor cells, and E—50% stainable tumor cells [21].

### 2.3. Hypoxia Response

The evaluation of immunohistochemical expression of HIF-1α (1:200, pH 6.0, Abcam, Cambridge, UK) was evaluated in preoperative and postoperative tissues, due to changes in the hypoxic state of the tumor during treatment. HIF-1 expression of HIF-1α was rated on the H score-scale, including intensity: 0—none, 1—weak, 2—moderate, 3—strong, and percentage of stained cells in each category (range: 0–300), as previously described [22,23].

### 2.4. Immune Infiltration

To estimate the number of TAMs samples (M1 and M2 classes) from patients before surgery were used. We have chosen CD163 (1:200, pH 9.0, Cell Marque, Rocklin, CA, USA), CD68KP (RTU, pH 9.0, Dako Agilent), and CD68 PG-M1 (RTU, pH 9.0, Dako Agilent, Santa Clara, CA, USA) to evaluate the number of TAMs. We scored TAM by counting the number of positive-staining macrophages per mm^2^: 0—none, 1—low, and 2—high.

### 2.5. Microvessel Density Analysis

Changes in IMVD were assessed by differences in CD105 (1:50, pH 9.0, Cell Signaling) and CD34 (RTU, pH 9.0, Dako Agilent) immunohistochemical expression. IMVDs were counted in five randomly selected fields at 200× magnification (>>6.9 mm^2^), and a total number of microvessels included a scoring scale: 1 (1–25), 2 (26–50), 3 (51–100), 4 (101–499) and 5 (>500) [24]. MVD was assessed based on immunohistochemical staining of CD34 (MVD/CD34) and CD105 (MVD/CD105), consistent with the method developed by Weidner [25]. The MVD was defined as the mean number of microvessels in the three most vascularized fields of view per 1 mm^2^.

### 2.6. DNA Damage Analysis

Analysis of changes in histone γH2AX expression was based on immunohistochemical staining of foci of the phosphorylated form of histone γH2AX (1:200, pH 9.0, Sigma Aldrich, St. Louis, MO, USA) in preoperative and postoperative STS tissues fixed on microscope slides. γH2AFX were rated on the H score scale, including intensity—0 none, 1 weak, 2 moderate, 3 strong—and percentage of stained cells in each category (range: 0–300) [22].

### 2.7. Statistical Analysis

Spearman rank correlations were measured to identify the correlation between biomarkers and PR. Statistical significance was established at *p* < 0.05. Statistical analysis was performed using R package version 3.6.3 software (R Foundation for Statistical Computing, Vienna, Austria).

## 3. Results

### 3.1. Characteristics of the Patient

Patients with marginally resectable and unresectable high-grade STS participated in the phase II clinical trial (NCT03651375) [16]. Due to the quantity and quality of the material, we analyzed the core biopsy tissue samples from 19 patients in this clinical trial. The enrolled patients included 11 patients with undifferentiated pleomorphic sarcoma (UPS), 4 patients with myxofibrosarcoma (MFS), 2 patients with leiomyosarcoma (LMS), 1 patient with pleomorphic liposarcoma (PLPS) and 1 patient with malignant peripheral nerve sheath tumor (MPNST). The patients were treated with 5 × 5 Gy RT combined with three cycles of AI chemotherapy, except for two patients who received one cycle of AI chemotherapy. One of these two patients did not receive RT. These patients were referred for limb amputation due to poor tolerance to chemotherapy. The characteristics of the patients are shown in Table 1.

### 3.2. Pathological Response

Good PR was noticed only in four cases (grades A = 1, B = 2, C = 1). In the other 15 cases, poor PR was reported (D = 11, E = 4). The summary of pathological evaluation was presented in Table 2.

### 3.3. Hypoxia Response

**Figure 1:**
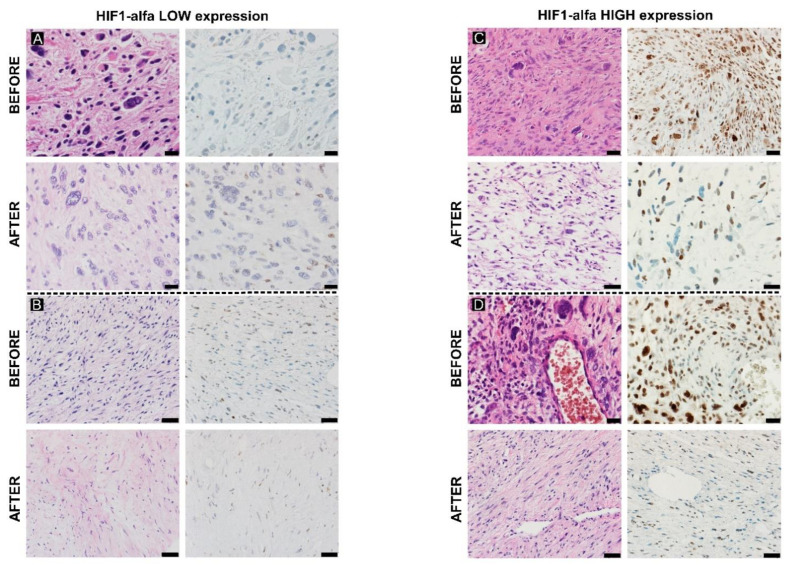
The differences in HIF-1α expression: the low expression before and after therapy in myxofibrosarcoma ((**A**), 400×) and leiomyosarcoma ((**B**), 400×), and high expression in undifferentiated pleomorphic sarcoma ((**C**), 400×) and malignant peripheral nerve sheath tumor ((**D**), 400×).

The expression of HIF-1α in the samples before surgery was higher than after surgery (Figure 1). The high initial expression of HIF-1α showed a negative correlation with PR (Spearman’s rho: −0.431), which means poor response to therapy. Additionally, decreased expression of HIF-1α after therapy confirmed the correlation between PR and HIF-1α expression.

### 3.4. Immune Infiltration

**Figure 2:**
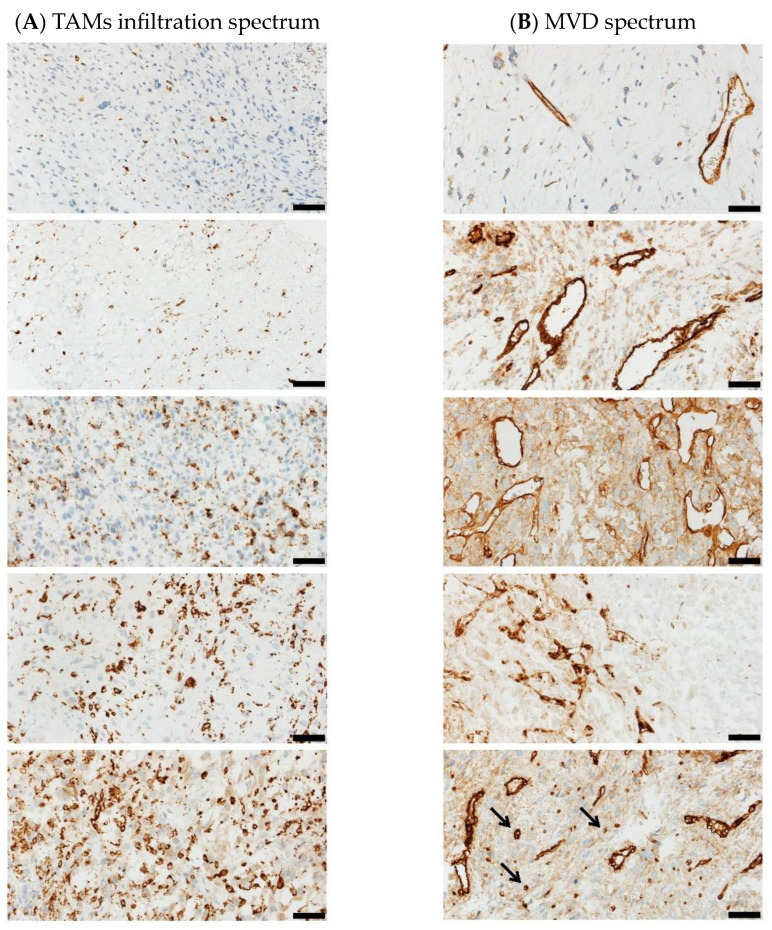
(**A**) Tumor macrophage infiltration showed the spectrum of immunohistochemical expression—the low (upper two images) and high (lower three images) density was seen (CD163, 200×); (**B**) the vessels density was evaluated according to 5th tier scale including the number of vessels per 1 mm^2^ (CD34, 200×).

In 15 samples, a high positive staining of TAM was detected (scores 2–4 cases, 3–4 cases, 4–2 cases, 5–5 cases) (Figure 2A). Low positive staining of the TAM was found in four cases (score 1–4 cases). There were no samples with a score of 0.

In addition, a high number of positive staining TAMs did not correlate with PR.

### 3.5. Microvessel Density Analysis

A total number of microvessels greater than 100 was observed in five cases (scores 4–2 cases, 5–3 cases). In four cases, the total number of microvessels reached 51–100 (scores 3–4 cases). The total number of microvessels under 50 was found in ten cases (scores 1–7 cases, 2–3 cases) (Figure 2B).

A high number of positive stainings of IMVD did not correlate with PR.

### 3.6. DNA Damage Analysis

**Figure 3:**
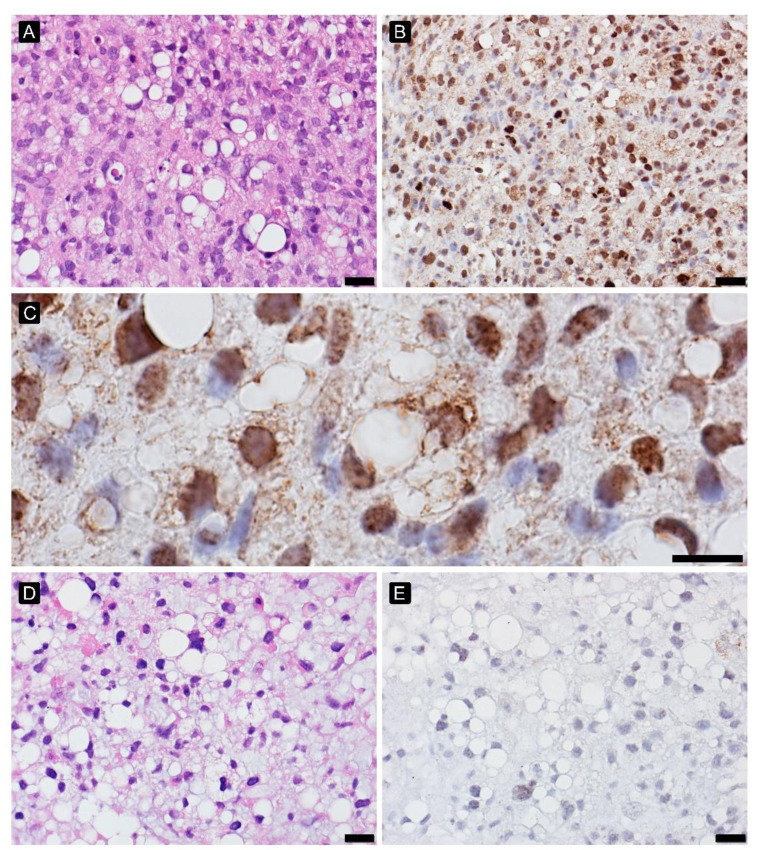
γH2AFX in myxoid liposarcoma high-grade before ((**A**)—HE, 200×, (**B**)—γH2AFX, 200× and (**C**)—γH2AFX, 600×) and after treatment ((**D**)—HE, 200×, (**E**)—γH2AFX, 200×).

**Figure 4:**
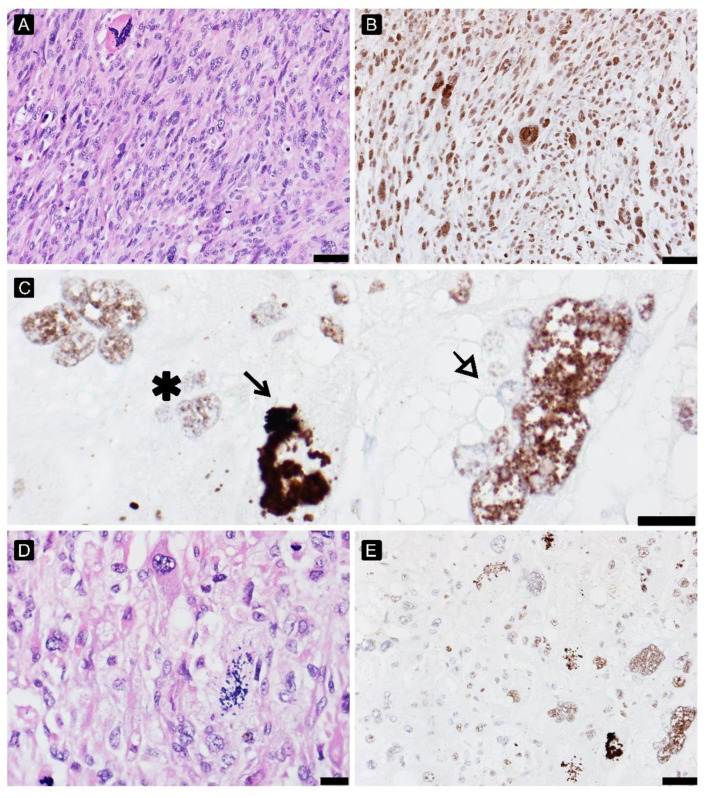
γH2AFX in undifferentiated pleomorphic sarcoma before ((**A**)—HE, 200×, (**B**)—γH2AFX) and after treatment ((**C**)—γH2AFX, 600×, asterix—“low grade” cells, arrow and white arrow—cells with apoptosis and “high grade” cells respectivelly with high γH2AFX expression level, (**D**)—HE, 200×, (**E**)—γH2AFX, 200×).

**Figure 5:**
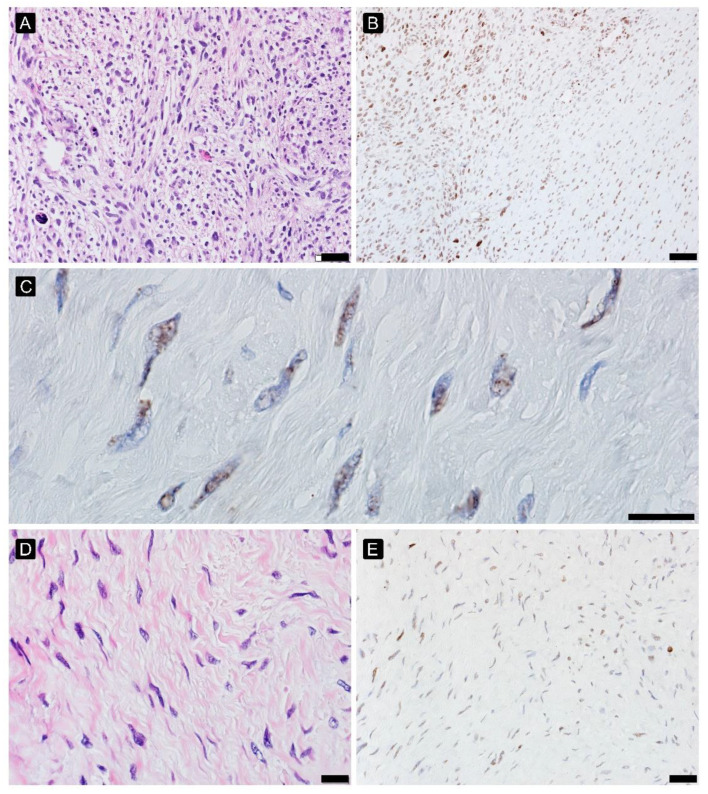
γH2AFX in malignant peripheral nerve sheath tumor ((**A**)—HE, 200×, (**B**)—γH2AFX) and after treatment ((**C**)—γH2AFX, 600× low expression, (**D**)—HE, 200×, (**E**)—γH2AFX, 200×).

High expression of γH2AFX before treatment positively correlated with PR (Spearman’s rho: 0.416), providing better PR. After treatment, the interpretation of γH2AFX was challenging: viable cells morphologically with a lower histological grade of malignancy have a lower expression, disintegrating during apoptosis and high grade pleomorphic cells showed higher expression. The γH2AFX expression profile in different histological patterns of STS was presented in Figure 3, Figure 4 and Figure 5.

## 4. Discussion

In our study, high expression of HIF-1α correlates with poor response to therapy. HIF-1α is a biomarker of the hypoxia microenvironment. This study shows an association between the expression of HIF-1α and the response to radiation therapy in STS patients. Hypoxic cells within a tumor limit the effectiveness of radiation therapy, requiring free oxygen to covert free radicals initiated by ionizing radiation to form DNA strand breaks. Measurement of the expression of the alpha subunit of HIF-1 may, therefore, be a predictor of response to treatment. High expression of HIF-1α may be a predictor of tumor radioresistance [26]. Most available studies on solid tumors, including breast [27], cervical [28], and brain cancer [29] studies, have shown significant overexpression of the HIF-1α protein regardless of oxygenation of the tumor tissue and its adverse prognostic effect on the RT used. The use of RT affects the release of reactive oxygen species (ROS), which inhibit the hydroxylation of the alpha subunit. Based on feedback, the HIF-1α protein accumulates, and pathways effect is the increase in the production of factors responsible for neo-angiogenesis, for example, vascular endothelial growth factors (VEGF) and fibroblast growth factors (FGFs) [30]. Overexpression of HIF-1α protein is known as an independent negative prognostic factor for STS. It was reported that patients with a strong or moderate expression of HIF-1α in STS have a significantly shorter OS in comparison with patients with a weak or no expression [31]. These results were confirmed by a recent analysis that high expression of HIF-1α is significantly correlated with shorter DFS (HR 2.05, *p* < 0.001), higher rate of metastasis (RR 3.21; *p* < 0.001), and shorter OS (HR 2.05, *p* < 0.001) in STS and bone sarcoma [32]. Moreover, downregulation of HIF-1α sensitizes sarcomas cells in vivo to radiation and decreases their clonogenic potential [26]. The nuclear accumulation of HIF-1α was shown in malignant peripheral nerve sheath tumor (MPNST) samples. HIF-1α accumulation was significantly correlated with poor prognosis. In an in vitro model, when HIF-1α was knockdown in MPNST cell lines, the cells’ proliferation was inhibited and cells underwent apoptosis. Inhibitor of Hsp90-HIF1α binding interaction in HIF1α’s N-terminus—chetomin treatment—also inhibited the growth of MPNST cells and induced their apoptosis [33]. In general, tumor hypoxia is associated with the aggressive biological behavior of the tumor, chemotherapy resistance, and treatment failure [34]. HIF-1α expression was recently correlated with radiotherapy response in breast cancer, oropharyngeal cancer [35], head and neck cancer [36], early esophageal cancer [37], and nasopharyngeal carcinomas [38] or cervical cancer [39]. The overexpression of HIF-1α is associated with a poor prognosis. HIF-1α signalling was also shown to be involved in drug resistance in multiple cancer types, including RCC, gastric, pancreatic, and gall bladder cancers. Overexpression of HIF-1α is correlated with poor prognoses and relapses during treatment [34,40].

Recent reports also emphasize the importance of TAMs. TAMs regulate the TME [3,4]. M1 is characterized by expression of main histocompatibility complex class II (MHC-II) cell surface receptor (HLA-DR), C-C Motif Chemokine Receptor 7 (CCR7, CD197), CD68, CD40, CD11c, CD80, and CD86 [11,12,13,14], while M2—CD163, CD209, CD206, CD204, CCL2, arginase-1 (ARG 1), and colony-stimulating factor receptor 1 (CSF1R) [3,11,12,13]. STS subtypes with the highest number of TAMs are dedifferentiated liposarcoma, leiomyosarcoma, undifferentiated pleomorphic sarcoma, and myxofibrosarcoma [41]. It was reported that high infiltration of TAMs correlates with poor overall survival (OS) and distant metastasis-free survival (DMFS) in STS and bone sarcomas [4,31,35]. In particular, high infiltration of CD68+ TAMs in the dedifferentiated chondrosarcoma with osteosarcoma compartment is correlated with short OS [31]. At the same time, in in embryonic rhabdomyosarcoma high levels of CD163^+^ are positively associated with survival [42], while in synovial sarcoma low CD163^+^ levels are associated with longer survival [43]. In a recent study of almost 200 STSs, infiltration of CD68+ macrophages was shown to be an independent biomarker of a higher risk of local recurrence in sarcomas [44,45]. Alteration in the density of CD163+ TAMs, CD68+ TAMs, and the CD163/CD68 ratio were reported in STS patients responding to neoadjuvant chemotherapy [46]. It was concurrently shown that programmed death cell receptor (PD-1), the ligand for PD-L1, and CD80-CD28/Cytotoxic T-Lymphocyte Associated Protein 4 (CTLA-4) ligand are expressed on TMAs. Such an expression profile enhances sarcoma tumor immune escape [2,4]. Moreover, TAMs may stimulate angiogenesis and metastases development [44,45], and their presence in most sarcomas (e.g., Ewing’s sarcoma, leiomyosarcoma) is significantly correlated with an unfavorable prognosis, including short OS [47,48].

Microvessel density that is measured most often by the number of CD105 and/or CD31-positive vessels/mm^2^ is a possible surrogate of angiogenesis [49,50]. This pathological feature of the tumors has shown an association with greater tumor aggressiveness [24]. Subsequently, in multiple types of cancer, including breast cancer, colon cancer, head and neck cancers, lung cancer, and prostate or ovarian cancer, it was shown that high IMVD correlates with poor prognosis in terms of survival, metastasis development, and therapy response [51,52]. We expected that assessing IMVD of STS at the time of diagnosis could give prognostic information for clinicians. In fact, angiogenic CD31 expression was correlated with chemotherapy resistance in sarcomas previously [53]. CD31 was also indicated to play a significant role in immune cell adhesion and integrin activation [53]. At the same time, CD34—an endothelial cell marker—is also considered as one of the main activators of angiogenesis in sarcomas, including sarcoma recurrence [54]. In a sarcoma study, median CD34 based IMVD was 44.6 for Ewing’s sarcoma, 39.7 for osteosarcomas, and 12.9 for chondrosarcomas [55]. Moreover, CD105 is also overexpressed in sarcomas in a HIF-1α dependent manner [56]. Higher levels of CD105-positive vessels correlate with high risk of death in RMS [57]. All the markers—CD31, CD34, and CD105—were shown as highly expressed within vessels with abnormal morphology, which further confirms the role of these molecules in tumor pathological growth [52]. Attempts to target CD105 and, as a result, the cells that express this protein in sarcoma tumor were promising in mice, but this was not successful approach in phase III trial in humans [58,59]. More research is needed to define the role of angiogenesis and anti-angiogenic therapies in sarcomas.

We used the expression of γH2AX to assess DNA double-strand breaks by detecting phosphorylated histone γH2AX. In our study, high expression of γH2AX correlated with favorable response to therapy. H2AX histones are a variant of histone H2A that possess a specific Ser-Gln motif in its C-terminal end. In response to DNA breakage, PI3 kinases phosphorylate H2AX histones [60]. Histone variant H2AX is phosphorylated at serine 139 due to double-strand breaks, and as a result gamma-H2AX is formed [61]. γH2AX is a highly specific and sensitive marker that indicates double-stranded DNA damage and genomic instability. Analysis of the frequency of histone γH2AX foci allows estimation of the radiation dose in the range of 0.1–5.0 Gy, because histone H2AX phosphorylation occurs after DNA damage and is associated with the repair of DNA double-strand breaks, which are characteristic of ionizing radiation [62]. Specifically, gamma-H2AX has already been studied in a variety of cancers including colon, breast, lung, ovarian, and cervix cancers. Although gamma-H2AX predicts survival in certain types of cancers (such as breast cancer and endometrial cancer), further research is needed to determine whether gamma-H2AX predicts survival in sarcomas [61]. γH2AX, combined with other DNA damage markers, may be more accurate in predicting survival. The combined expression of PARP1, γH2AX, BRCA1, and BRCA2 was an independent prognostic predictor of shorter disease-specific survival (DSS) and event-free survival (EFS) in the study of 112 STSs [63]. The major limitation in sarcoma research is the low number of cases studied due to the epidemiology of the disease. We expect to recruit more patients in future biomarkers trials if studied cohort characteristics allow recruitment of higher number of patients for pre-planned therapy approach.

## 5. Conclusions

HIF-1α and γH2AFX are potential biomarkers for STS neoadjuvant treatment response prediction. Further research is needed to provide validation of these markers in an independent cohort. Biomarker studies should be incorporated in the clinical trial design to improve care for patients with metastatic sarcoma in the future. Hypoxia and DNA damage response are pathways involved in sarcoma cells response to multidisciplinary treatment and pathway-wide studies could provide additional biomarkers potentially increasing prediction specificity.

## Figures and Tables

**Table 1:** Characteristics of the patient at the time of diagnosis.

Characteristics	Value, *n* (%)
Age at diagnosis	
Median	58
Range	32–75
Gender	
Female	8 (42.11%)
Male	11 (57.9%)
Tumor pathology	
Undifferentiated pleomorphic sarcoma (UPS)	11 (57.9%)
Myxofibrosarcoma (MFS)	4 (21.05%)
Leiomyosarcoma (LMS)	2 (10.53%)
Pleomorphic liposarcoma (PLPS)	1 (5.26%)
Malignant peripheral nerve sheath tumor (MPNST)	1 (5.26%)
Tumor site	
Trunk wall	2 (10.53%)
Arm/shoulder	1 (5.26%)
Thigh/buttock	2 (10.53%)
Calf	14 (73.68%)
Grade	
G2	8 (42.11%)
G3	11 (57.9%)
Largest tumor dimension	
5–10 cm	3 (15.79%)
>10–15 cm	8 (42.11%)
>15–20 cm	7 (36.84%)
>20–25 cm	1 (5.26%)
>30 cm	1 (5.26%)
Given doxorubicin-ifosfamide chemotherapy	
1 cycle	19 (100%)
2 cycles	17 (89.47%)
3 cycles	17 (89.47%)
Completed radiotherapy	
Yes	18 (94.74%)
No	1 (5.26%)

**Table 2:** The summary of pathological evaluation HIF-1α, TAM, IMVD, γH2AFX and necrosis/response score after treatment.

	Before Treatment	After Treatment
**HIF-1α**	176 (range 90–240)	122 (range 60–180)
**TAM**	1–4 (21.05%); 2–4 (21.05%); 3–4 (21.05%); 4–2 (10.53%); 5–5 (26.32%)	
**IMVD**	1–7 (36.84%); 2–3 (15.79%); 3–4 (21.05%); 4–2 (10.53%); 5–3 (15.79%)	
**γH2AFX**	216 (range 140–300)	
**Necrosis**		67% (range 0–100%)
**Response score**		A—1 (5.26%); B—2 (10.53%); C—1 (5.26%); D—11 (57.89%); E—4 (21.05%)

## Data Availability

Data may be obtained from PI of the study upon scientific collaboration request and DTA agreement.
